# Transcriptional response to cardiac injury in the zebrafish: systematic identification of genes with highly concordant activity across *in vivo* models

**DOI:** 10.1186/1471-2164-15-852

**Published:** 2014-10-03

**Authors:** Sophie Rodius, Petr V Nazarov, Isabel A Nepomuceno-Chamorro, Céline Jeanty, Juan Manuel González-Rosa, Mark Ibberson, Ricardo M Benites da Costa, Ioannis Xenarios, Nadia Mercader, Francisco Azuaje

**Affiliations:** NorLux Neuro-Oncology Laboratory, CRP-Santé, Luxembourg, Luxembourg; Genomics Research Unit, CRP-Santé, Luxembourg, Luxembourg; Departamento Lenguajes y Sistemas Informáticos, Universidad de Sevilla, Seville, Spain; Cardiovascular Research Center, Massachusetts General Hospital and Harvard Medical School, Boston, USA; Vital-IT Systems Biology/Medicine Department, SIB Swiss Institute of Bioinformatics, Lausanne, Switzerland; Center for Integrative Genomics, University of Lausanne, Lausanne, Switzerland; Department of Biochemistry, University of Geneva, Geneva, Switzerland; Department of Cardiovascular Development and Repair, Centro Nacional de Investigaciones Cardiovasculares Carlos III, CNIC, Madrid, Spain

**Keywords:** Myocardial infarction, Zebrafish, Ventricular amputation, Ventricular cryoinjury, Heart regeneration, Transcriptional responses, Transcriptional association networks

## Abstract

**Background:**

Zebrafish is a clinically-relevant model of heart regeneration. Unlike mammals, it has a remarkable heart repair capacity after injury, and promises novel translational applications. Amputation and cryoinjury models are key research tools for understanding injury response and regeneration *in vivo*. An understanding of the transcriptional responses following injury is needed to identify key players of heart tissue repair, as well as potential targets for boosting this property in humans.

**Results:**

We investigated amputation and cryoinjury *in vivo* models of heart damage in the zebrafish through unbiased, integrative analyses of independent molecular datasets. To detect genes with potential biological roles, we derived computational prediction models with microarray data from heart amputation experiments. We focused on a top-ranked set of genes highly activated in the early post-injury stage, whose activity was further verified in independent microarray datasets. Next, we performed independent validations of expression responses with qPCR in a cryoinjury model. Across *in vivo* models, the top candidates showed highly concordant responses at 1 and 3 days post-injury, which highlights the predictive power of our analysis strategies and the possible biological relevance of these genes. Top candidates are significantly involved in cell fate specification and differentiation, and include heart failure markers such as periostin, as well as potential new targets for heart regeneration. For example, *ptgis* and *ca2* were overexpressed, while *usp2a*, a regulator of the p53 pathway, was down-regulated in our *in vivo* models. Interestingly, a high activity of *ptgis* and *ca2* has been previously observed in failing hearts from rats and humans.

**Conclusions:**

We identified genes with potential critical roles in the response to cardiac damage in the zebrafish. Their transcriptional activities are reproducible in different *in vivo* models of cardiac injury.

**Electronic supplementary material:**

The online version of this article (doi:10.1186/1471-2164-15-852) contains supplementary material, which is available to authorized users.

## Background

The zebrafish (*Danio rerio*) has the capacity to regenerate its heart after undergoing severe injury [1, 2]. This capability has been shown in adult zebrafish using different *in vivo* models of cardiac damage, and involves the generation of new cardiomyocytes from existing ones located near the injury site [3, 4]. Thus, the zebrafish represents a compelling model to study heart injury and regeneration with potential clinical impact. In the long-term, this will be crucial to address a major world-wide public health problem: heart attacks (myocardial infarction) followed by heart failure [5]. The latter is a consequence of the incapacity of the human heart to replace the lost cardiomyocytes by newly formed myocardium, instead forming an irreversible fibrotic scar [6, 7].

Identification of the cellular and molecular mechanisms of zebrafish heart regeneration might thus allow to find new therapeutic approaches for the treatment of myocardial infarction in humans. Despite obvious differences between teleosts and mammals, the sequencing of the zebrafish genome revealed that about 70% of human genes have at least one zebrafish orthologue [8]. A large amount of genetic screens have already been performed in zebrafish in order to identify genes involved in cardiac development and regeneration. Random mutagenesis, creation of transgenic strains using inducible genetic approaches or targeted gene inactivation using morpholinos have led to generation of different models of human cardiac disorders [9–16], whose number will undoubtedly be expanded by new genome editing techniques, such as Crisp/Cas9 and TALEN. Moreover, the external development of the zebrafish embryo and its transparency allows direct microscopic observation of cardiovascular structures without invasive instrumentation. Coupled to its ability to survive for several days without functional cardiovascular system, catching oxygen by passive diffusion [17], these attributes make zebrafish particularly suitable to study the phenotype of cardiovascular diseases. Besides, response to drugs is well conserved between fish and mammals [18], making zebrafish a widely used model for toxicological analysis and to study the possible cardiac effects of chemical compounds.

Different techniques have been set up to study the remarkable cardiac regeneration ability of adult zebrafish. Coronary artery ligation, mimicking ischemic injury that leads to myocardial infarction in human, is commonly used as model of myocardial infarction in mice. However, the size of the ventricle (around 1 mm^3^) renders this kind of injury almost impossible to achieve in zebrafish. Consequently, different alternative models have been used to study myocardial infarction in adult zebrafish: ventricular amputation, cryoinjury and cell-type specific ablation [19, 20]. The oldest and most widely used technique is ventricular amputation, which consists of removing about 20% of the apex of the ventricle by surgical resection. Following surgery, the apex is sealed by a clot of erythrocytes, further replaced by fibrin. Two months post-injury, the clot is not replaced by scar tissue but by cardiac muscle formed by cardiomyocytes proliferation, restoring the contractile properties of the heart [21]. More recently, the cryocauterization model, which is already used in mouse [22], has been applied to zebrafish. In this technique, a cryoprobe or dry ice is used to freeze 15 to 25% of the ventricle [23–27]. Following cryocauterization, blood accumulates in the infarct area and massive cell death can be observed at the injury site. A large fibrotic scar is formed and further replaced by cardiac tissue following cardiomyocytes proliferation. Total recovery is longer than for ventricular amputation (up to 130 days [23]), which may be due to the extra time needed to remove the necrotic tissue and heal the scar. Finally, a genetic cell ablation model based on the Cre-lox strategy was set up to specifically kill cardiomyocytes in an inducible manner throughout the heart [28]. This technique allows destruction of 60% of cardiomyocytes and promotes signs of cardiac failure. Upon cell death, inflammatory cells are recruited, endocardial and epicardial cells are activated and cardiomyocytes de-differentiation and proliferation are enhanced, leading to muscle regeneration by 1 month after injury. Among these cardiac regeneration models, cryoinjury is perhaps the most relevant for translational biomedical research. It resembles what is observed in the human heart following myocardial infarction, including the fact that the injury affects all cardiac cell types and promotes massive cell death and the formation of a fibrotic scar.

The analysis of transcriptional responses following injury is a powerful approach to identifying candidate drivers of heart repair and regeneration, which may represent potential targets for triggering or boosting cardiomyocyte renewal in mice and humans [29, 30]. Mainly using amputation and genetic ablation models, several studies have characterized differential expression responses at different times after injury based on microarrays or *in situ* hybridization techniques [31]. These techniques in combination with generation of transgenic zebrafish strains and protein concentration analysis have demonstrated the regulatory roles of biological pathways implicated in cell proliferation and differentiation, such as retinoic acid [32], platelet-derived growth factor [31, 33], sonic hedgehog, Insulin-like growth factor, Transforming growth factor beta [34, 35], Jak1/Stat3 [30] and Notch signalling [36].

Despite the insights derived from previous research, there is still a need to: a. apply biologically unbiased approaches to dissecting transcriptional responses after injury, and b. investigate the validity of relevant findings across different *in vivo* models. The former involves the characterization of gene expression patterns beyond the identification of genes differentially expressed. The latter is important not only to reconcile different *in vivo* models that are promising for translational research, but also to identify genes with post-injury transcriptional behaviours that are highly robust regardless of injury model. Previous investigations conducted by our team and elsewhere have demonstrated that the application of network-based approaches to gene expression analysis can: 1. enhance systems-level understanding of key biological states; and 2. identify candidate markers of cardiac damage, which are not only accurate for sample classification but also reproducible in independent datasets and model organisms.

Here, our main objective is to identify genes exhibiting important transcriptional perturbations after heart injury in the zebrafish, which are both biologically meaningful and reproducible in independent *in vivo* models.

To address this question, we applied different computational analysis strategies with an emphasis on network-based approaches, which identify significant gene-gene associations from the expression data. Our key premise was that genes with prominent patterns of “transcriptional connectivity” in such networks represent genes with potential critical roles in the response to injury. Here, we show that across *in vivo* models, a set of top candidates showed highly concordant responses at 1 and 3 days post-injury (dpi). This set of computationally predicted candidates are significantly associated with processes relevant to cell fate specification and differentiation. They include genes with suspected functional roles in heart regeneration, as well as novel genes that may exercise influential roles in the early stages of injury response.

## Results

### Overview of discovery framework

**Figure 1:**
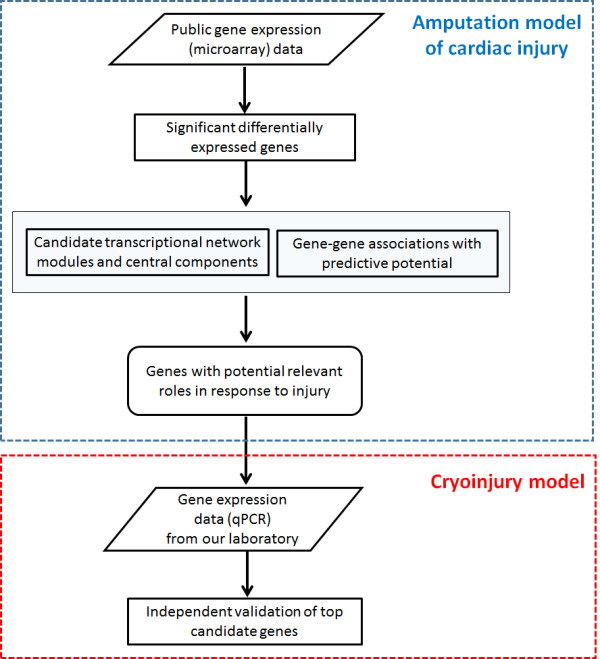
**Overview of analytical and modelling workflow.**

Figure 1 summarises our analytical and modelling workflow. First, we analysed a published dataset that consists of gene expression measurements of sham-operated and injured hearts at 1, 3, 5 and 7 days post-amputation [29]. From now on, we will refer to this dataset as the *model derivation dataset*. We detected genes significantly differentially expressed across all samples, and selected them for further analysis. We then applied three published computational approaches to generating and analysing gene co-expression networks: Clustering with Overlapping Neighborhood Expansion (ClusterONE) [37], inference of gene regression networks with model trees (RegNet) [38], and the Weighted Gene Co-Expression Network (WGCNA) algorithm [39]. This allowed us to identify: networks clusters, highly connected genes, and genes that can be used as estimators of the expression values of other genes in the dataset (Methods). We independently performed these analyses, focused on (statistically) top-ranked results, and selected a list of *candidate genes* for further computational and experimental analysis. In the validation phase, we implemented the cryoinjury model at our laboratory and measured the expression of the candidate genes by RT-qPCR (Methods). From now on, we will refer to this dataset as the *model validation dataset*. In the network models reported here, nodes and edges represent genes and between-gene expression correlations respectively.

### Data pre-processing and differential expression analysis

Processed Affymetrix GeneChip Zebrafish microarray data were downloaded from ArrayExpress (E-GEOD-17993) resulting of 15617 features with 10773 unique gene symbol [29]. Model derivation dataset consists of 15 microarrays: 3 replicates over 5 time points (control, 1, 3, 5 and 7 day after operation). Pre-filtering of features decreased the size of dataset to 7182 features corresponding to 5840 genes with unique gene symbol. Limma analysis resulted in 3403 unique annotated genes variable across the time points with FDR < 0.05, 88 genes at FDR ≤ 0.0001. The number of differentially expressed genes (FDR < 0.05) after applying 2-condition contrasts varied in time: 1907, 2349, 1914, and 330 annotated genes for control vs. 1, 3, 5 and 7 days respectively. Thus the highest dysregulation of the transcriptome was observed at the 3^rd^ day after injury, while other important events required for heart regeneration occur in the days following injury.

A second dataset comes from independent work by Kizil et al. [40] (E-MEXP-1239). Here, 11 arrays were used: 4 controls replicates, 1 replicate for each of 6 hours, 12 hours and 1 day, 2 replicates for 3 and 5 days. We were able to identify only 34 differentially expressed genes with FDR < 0.05 in this dataset.

### Identification of potentially relevant genes with network-based approaches

**Figure 2:**
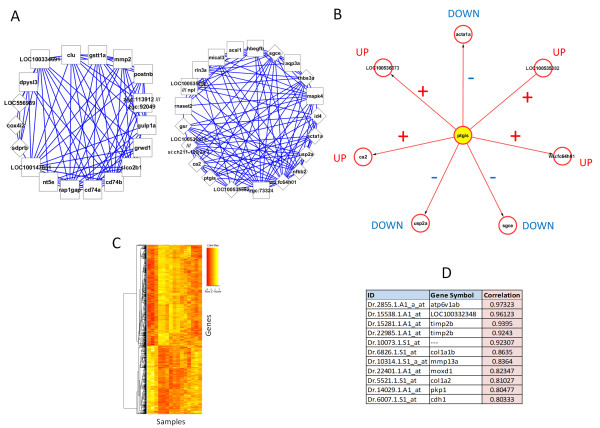
**Computational models generated from public data.** Different approaches highlight genes and associations with potential influential roles in response to injury. **A**. Top network modules identified with the ClusterONE algorithm [37]. Network nodes shown as rectangles: genes with exclusive membership in the modules shown, diamonds: genes with multiple module membership. Edges represent co-expression associations. **B**. Top candidate regulatory circuit inferred with the RegNet algorithm [38]. Edges represent gene co-expression association, with arrows defining the direction of the association between *ptgis* and the other genes according to linear regression models. The latter are shown for each gene-gene association. **C**. Snapshot of network module identified with the WGCNA algorithm, which contains genes involved in A and B, including *ptgis* (higher resolution image in Additional file 2). **D**. List of genes with significant concordant expression values between our model derivation dataset [29] and a dataset obtained from an independent study based on amputation model [40]. Between-dataset correlation values are shown.

After computing between-gene correlations among top-differentially expressed genes in the derivation dataset (88 differentially expressed genes at FDR ≤ 0.0001), we generated a gene co-expression network. This network consisted of 3828 edges representing gene co-expression relationships estimated with the Pearson correlation coefficient, *r*. To reduce the number of potential spurious associations and facilitate visualizations, we filtered out edges with │*r*│ < 0.95. To verify the potential statistical relevance of these associations, we estimated their maximal information coefficients (MICs) and confirmed the high co-expression relationships of these genes (MIC > 0.95). The resulting network included 86 nodes and 346 edges. A dynamic visualization of this network at different time points and their underlying data are available in Additional file 1. The ClusterONE algorithm identified two highly statistically significant network modules (Figure 2A): one consisting of 18 nodes and 79 edges (P = 1.4E-7), and another consisting of 21 nodes and 107 edges (P = 3.3E-7). The first module contained *postnb* (periostin b). Periostin is a known fibroblast marker, which is found in the scar tissue in both mouse and zebrafish [41–46]. While its role in myocardial proliferation remains to be fully elucidated, interestingly, its expression precedes heart regeneration in the zebrafish [46], which is consistent with our findings in two *in vivo* injury models. The second module also includes potentially interesting genes, such as *usp2a* (Ubiquitin Specific Peptidase 2) and *mapk4* (Mitogen-Activated Protein Kinase 4), which are implicated in cell death and proliferation signalling pathways [47, 48].

Independently, we applied the RegNet algorithm on the reduced dataset (88 genes at FDR ≤ 0.0001) to extract transcriptional association networks (Methods). This modelling technique specifies mathematical relationships between the genes based on linear regression models. Such associations are graphically represented with directed edges to indicate which genes can be used as reliable estimators of the expression values of other genes in the dataset (Figure 2B). RegNet identified a statistically significant network (alpha = 0.05, which is set to control the FDR using Benjamin-Yekutieli procedure, and theta = 30%, which is set as a relative error threshold) containing 8 nodes and 7 edges (Figure 2B), which overlaps with the modules identified previously (Figure 2A). In this network, *ptgis* (Prostaglandin I2 synthase) is shown as the central node, and its expression data are correlated (positively and negatively) with the other genes in the network. This allows a qualitative labelling of each network edge with the direction of the observed gene-gene associations. For example, *ptgis* is negatively correlated with *acta1a* (upper gene in Figure 2B). This means that the expression values of these genes have opposite responses in the dataset. Therefore, we can use the sign of these associations to tag the direction of the relationship between *ptgis* and the other genes: UP (positive association) and DOWN (negative association). These results highlight *ptgis* as a candidate relevant gene based on its potential to estimate the expression response of other genes in the dataset.

To further assess the potential predictive relevance and robustness of these findings, we applied the WGCNA algorithm to a larger version of the dataset: 3403 differentially expressed genes at FDR ≤ 0.05, and with gene associations calculated with the Spearman correlation coefficient. As in the results from ClusterONE and RegNet, WGCNA found a strong association between *ptgis* and *acta1a* within the same network module consisting of 1013 genes (Figure 2C, high resolution version in Additional file 2). For *ptgis*, WGCNA reports a very high module membership (MM) score of 0.95 (P = 2.3E-08), which also highlights the prominent centrality of *ptgis* within this highly interconnected module. In this module *acta1a* shows a MM = -0.9 (P = 4.9E-06) that not only indicates its negative correlation with *ptgis*, but also its multiple strong connections with the other members of this module. We repeated this analysis on a network generated with Pearson correlations, and consistent associations between *ptgis* (MM = 0.9) and *acta1a* (MM = -0.87) were detected within the same, though smaller, module (616 genes). These results gave us further evidence of the potential relevance of the association between *ptgis* and *acta1a*.

As we are interested in identifying genes with robust and reproducible transcriptional responses, we complemented our findings with an analysis of concordant gene expression patterns in relation to an independent dataset [40]. As in our model derivation dataset, Kizil et al.’s dataset was obtained from microarray measurements from a heart amputation model in zebrafish. Unlike our model derivation dataset, Kizil et al.’s dataset exhibited a reduced set of significantly differentially expressed genes (34 genes differentially expressed at FDR < 0.05) across control and post-injury samples. We searched for highly concordant expression response between these two datasets (Pearson correlation > 0.8) at control and days 1, 3 and 5 post-injury. Figure 2D displays the resulting list of genes with strong between-dataset expression correlations. Although none of these genes were highlighted as potentially relevant by our network-based approaches, they deserved further investigation based on their highly robust behaviour in independent studies. Moreover, we note that *mmp13a* (matrix metalloproteinase 13a) and LOC100332348 (an uncharacterized protein coding gene) were found together with *ptgis* and *acta1a* in the same WGCNA module.

### Selection of candidate genes for independent experimental validation

**Figure 3:**
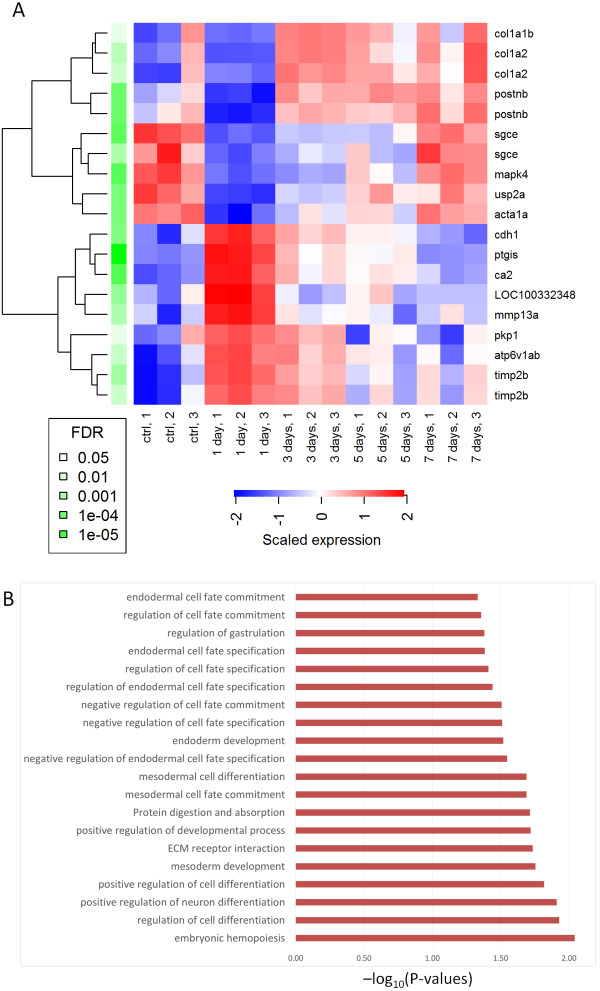
**Candidate genes: their transcriptional responses and potential functional implication. A**: Summary of expression patterns displayed by set of top candidates. Level of the statistical significance of their expression changes across samples are shown with their corresponding FDR values. **B**: Statistically significant associations between candidate genes and biological processes detected by IMP (FDR-corrected P-values < 0.05) [49]. Plot shows –log_10_(P-values).

Next, we selected a list of genes for independent experimental validation at our laboratory. Among the genes that were identified above as potentially relevant, we decided to focus our attention on 15 genes that met the following criteria: a. genes that were highlighted by at least one of the techniques reported above, b. genes that have not been functionally characterized in the zebrafish or associated with its heart regeneration capacity, c. genes with unambiguous and accurate matching qPCR primers for the zebrafish genome. Additionally, we included *postnb* because of its known activity in the early stages of heart regeneration in zebrafish. Figure 3A shows the list of selected candidate genes, and a summary of their expression responses in the model derivation dataset. In this visualisation we also plot the probe-specific expression levels for those genes with multiple microarray probes in the model derivation dataset. Such cases show high expression consistency within the dataset.

After selecting the candidate genes, we detected statistically significant associations between this gene set and cellular processes with the IMP (Integrative Multi-species Prediction) system [49]. IMP identified 20 significant associations (FDR-corrected P-values < 0.05) between our candidate genes and diverse cellular processes relevant or related to cell proliferation, differentiation and cell-cell interactions (Figure 3B). Some are specifically related to mesoderm and endoderm development. Interestingly, while cardiomyocytes derive from the mesoderm, factors secreted by the endoderm are necessary to induce differentiation of the mesoderm in heart tissue during embryonic development [50–54]. We might speculate that the crosstalk between endodermal and mesodermal cells that allows to regulate cardiogenesis during vertebrate embryonic development may be re-activated following myocardial infarction in adult to promote cardiac regeneration. These computational predictions, made by IMP based on the analysis of diverse independent molecular datasets, further demonstrate the potential relevance of our candidate genes.

Also we assessed the transcriptional concordance of our candidate genes in relation to the dataset by Lien et al.’s [30]. We detected a high positive correlation in their responses (log2FC) at 3 dpi (Pearson correlation, *r* = 0.955). Moreover, there is a positive linear correlation at day 7 (*r* = 0.636). These analyses provide further evidence of the robust responses of our candidate genes across time and *in vivo* injury models. It is also worth noting that the high concordance of our candidate genes at 3 dpi was detected despite the fact that Lien et al.’s dataset shows a much smaller set of significantly differentially expressed genes than our derivation dataset at 3dpi (326 vs. 1123 genes, FDR < 0.01).

### Independent validation with a cryoinjury model

In order to independently validate, *in vivo*, the results obtained from the analysis of public microarray data, we induced myocardial infarction in zebrafish using the cryoinjury technique. Experiments were performed on WIK wild-type fish aged 10–11 months. For sham surgery, we opened the body and the pericardial sac, without touching the ventricle, as previously described [29]. Animals were sacrificed and hearts were recovered 1 or 3 days post-surgery. Three independent experiments comparing gene expression in sham vs. cryoinjured animals were performed. In each experiment, the ventricle was separated from the bulbus and the atrium following dissection, and 3 to 4 ventricles were pooled per sample in order to perform the qPCR validation. To visualize the cryoinjured area, we performed immunofluorescence stainings of the ventricle of sham and cryoinjured animals. Hearts of sham-operated animals expressed tropomyosin in the whole ventricle and in the atrium, while tropomyosin staining appeared very weak in the injured area one day post-injury (Additional file 3). Furthermore, while no apoptotic cells were visible in sham-operated hearts, many TUNEL-positive cells were detected in the lesioned area at day-1 post-injury (Additional file 3). These results confirmed massive cell death of cardiomyocytes in the injured area at day-1 following cryoinjury.

**Figure 4:**
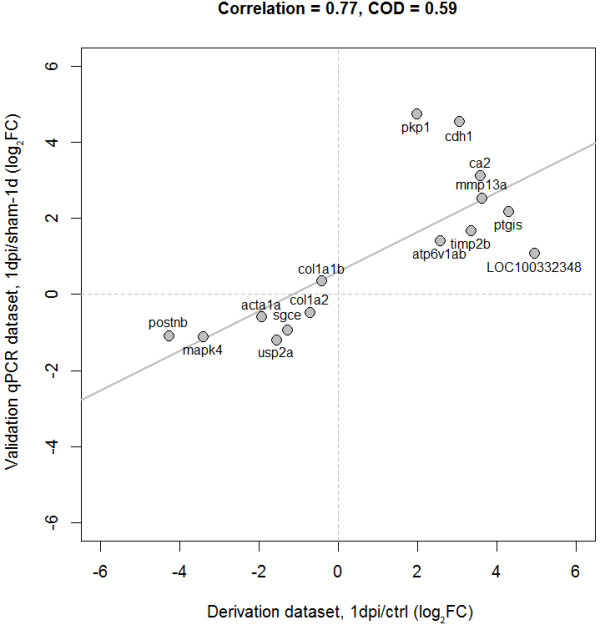
**Candidate genes show significant concordance of expression patterns across independent** ***in vivo*** **models at day-1 post-injury.** Log2-transformed fold-changes (FC) observed at day-1 (in relation to controls). Independent observations show strong positive linear relationships (Pearson correlation coefficient: 0.77, coefficient of determination: 0.59).

**Figure 5:**
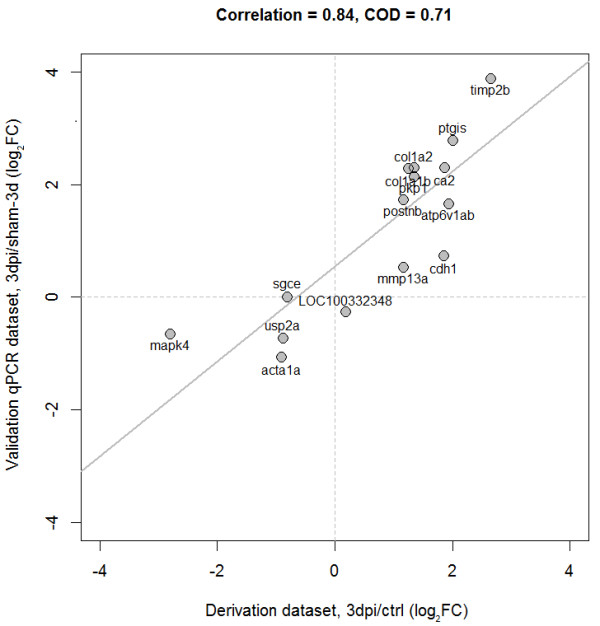
**Candidate genes show significant concordance of expression patterns across independent** ***in vivo*** **models at day-3 post-injury.** Log2-transformed fold-changes (FC) observed at day-3 (in relation to controls). Independent observations show strong positive linear relationships (Pearson correlation coefficient: 0.84, coefficient of determination: 0.71).

We measured the expression of the candidate genes with qPCR 1 and 3 days after cryoinjury and from sham operated animals (3 injured vs. 3 sham samples for each time point). We assessed whether qPCR expression responses were consistent with those observed in the amputation model. We found a high concordance between the expression responses at day-1 from the model derivation and validation datasets (Figure 4). More specifically, strong positive linear correlations were detected between their (log2-transformed) expression fold-changes at day-1 (Pearson’s correlation, *r* = 0.77, P = 0.0008). Our validation experiments at day-3 also showed that the transcriptional response of our candidate genes is highly concordant between independent datasets and *in vivo* models. As before, we observed a high positive correlation between the derivation and validation datasets (*r* = 0.84, Figure 5).

Next, we tested the capacity of the RegNet-derived network (Figure 2B) to reproduce its predicted associations in our cryoinjury model. From this network, the following candidate genes were included in the independent qPCR dataset: *ptgis*, *ca2*, *usp2a*, *sgce* and *acta1*. Thus, we tested whether the following expression associations were preserved:

A. *ptgis* and *ca2* (positive association)
B. *ptgis* and *usp2a* (negative association)
C. *ptgis* and *sgce* (negative association)
D. *ptgis* and *acta1* (negative association)

**Figure 6:**
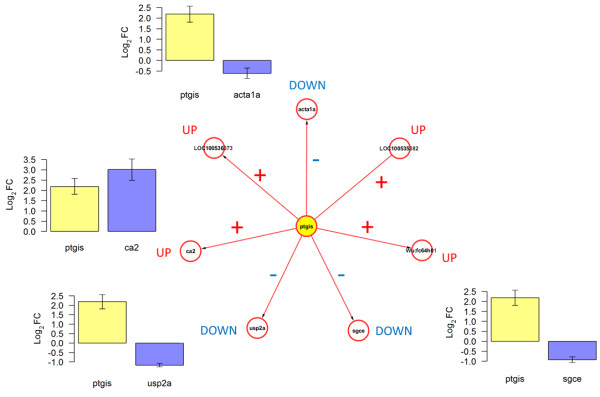
**Predicted relationships between** ***ptgis*** **and its candidate regulated genes are reproduced in the cryoinjury model at day-1 post-injury.** Plots show the Log2FC observed in independent qPCR data validation at day-1 (in relation to sham-1d), which are qualitatively consistent with the associations predicted by the computational model inferred from public data (Figure 2B). Error bars: Standard errors based on expression variability of 3 biological replicates for experimental and control conditions.

**Figure 7:**
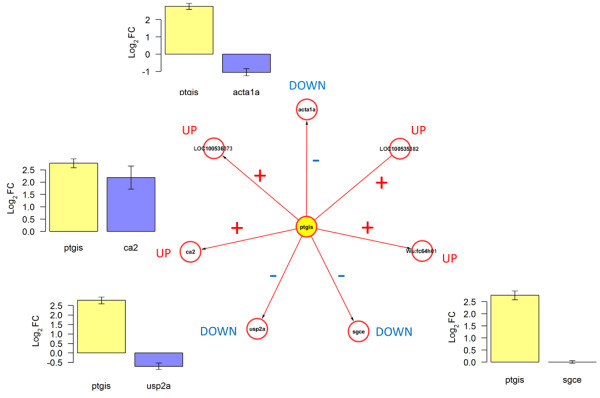
**Predicted relationships between** ***ptgis*** **and its candidate regulated genes are reproduced in the cryoinjury model at day-3 post-injury.** Plots show the Log2FC observed in independent qPCR data validation at day-3 (in relation to sham-3d). Error bars: Standard errors based on expression variability of 3 biological replicates for experimental and control conditions.

Figures 6 and 7 illustrate the results from this independent test, and indicate a good reproducibility of predicted associations at day-1 and -3. This network model correctly described the qualitative gene-gene (fold-change) relationships observed in the validation dataset. The only exception to the latter was the response of *sgce*, which showed a Log2FC = 0.003, instead of the negative value predicted. An alternative visualization of the changes in (log2) expression of these genes is available in Additional file 4. This is further evidence of the predictive potential of this network model, and of the putative relevance of *ptgis* as a transcriptional control component in the early response to heart injury in both amputation and cryoinjury models.

## Discussion

The main contributions of our research are: a. based on the combination of different computational approaches and information resources, we have identified genes with potential functional roles in the early response to heart injury in the zebrafish; b. we have demonstrated the potential predictive value of network-based approaches to systematically dissect complex transcriptional phenomena; and c. we have shown that the identified gene expression responses and their relationships are reproducible in two *in vivo* models of heart injury: amputation and cryoinjury.

A central objective of our study was to show transcriptional response concordance between two *in vivo* models of cardiac injury: starting with the traditional one (resection) and then validating the findings on a more recent, clinically-motivated one (cryoinjury). In particular, we aimed to demonstrate that the integrative bioinformatics analysis of public datasets could result in the identification of novel, biologically interesting candidate genes, whose responses to injury are sufficiently robust or reproducible across datasets and *in vivo* models.

The top-15 candidate genes identified in our analysis are mostly involved in cell fate specification and differentiation. Among them, periostin, which regulates cardiac homeostasis, is a well-known marker of heart failure [55]. Interestingly, among the 8 genes highlighted with the RegNet algorithm, some of them were previously associated with cardiac hypertrophy while others are still of unknown function in the heart. The central node, *ptgis*, codes for prostaglandin I2 (prostacyclin) synthase, prostacyclin acting as a vasodilator and inhibiting platelet aggregation. *Ptgis*’s up-regulation was recently shown to be associated with cardiac hypertrophy and heart failure in rat [56]. Furthermore, recent research has established connections between prostaglandins and regeneration of the brain in zebrafish [57] and the heart in mice [58].

Likewise, *ca2* mRNA and protein expression was also found to increase during the progression of ventricular hypertrophy in human [59], suggesting that *ca2* could be a prognostic marker of heart failure. *Acta1*, encoding skeletal muscle alpha actin, is one of the major components of the contractile apparatus. While the down-regulation of *acta1a* in our experiments might only reflect a decrease in the number of cardiomyocytes upon injury, it could also represent a *bona fide* downregulation of the gene. Indeed, mutations in the *acta1* gene in humans, impairing muscle cells’ contraction, are implicated in multiple myopathies [60, 61]. Among them, nemalin myopathy is the most common disorder linked to mutation in *acta1* gene, and has been occasionally associated with hypertrophic cardiomyopathy [62–64]. *Acta1* expression was also found to be deregulated in a screen measuring cardiac gene expression in patients suffering from hypertrophic cardiomyopathy [65]. Moreover, sarcoglycans are components of the dystrophin – glycoprotein complex, which link the actin cytoskeleton to the extracellular matrix in cardiac and skeletal muscle cells. *Sgce* codes for epsilon sarcoglycan and is highly homologous to alpha sarcoglycan (*sgca*). While *sgce* null mice do not display any cardiac phenotype, *sgca/sgce* double mutant mice develop dilated cardiomyopathy [66], suggesting a role for *sgce* in regulating proper cardiac function. Interestingly, expression of the ubiquitin-specific protease 2a is down-regulated in our *in vivo* models of myocardial infarction. While the role of USP2a has been investigated in cancers, where it has been shown to regulate p53 pathway or EGFR degradation [67, 68], its cardiac function is still unknown. A recent investigation showed that a tight control of p53 regulation is a prerequisite for dedifferentiation and proliferation during limb regeneration in the newt [69].

We are aware that additional independent experimental analyses will be required to confirm or characterize the potential functional role of our candidate genes. Also our study is limited by a relatively small amount of data. To address these constraints and facilitate further investigations, we have put emphasis on the reduction of possible false positive relations. This was done by defining relatively stringent criteria of statistical significance, and by applying multiple computational approaches that were capable to highlight shared associations among the candidate genes.

Although our results indicate that the candidate genes are both potentially novel and biologically meaningful players, at this stage we do not claim that they include “regulators” of cardiac regeneration. The latter will require further analysis and functional investigations.

Also, as all models, the cryoinjury technique used in this investigation does not perfectly reflect what actually happens during myocardial infarction in humans. Indeed, the amputation technique does not promote either cell apoptosis or formation of a fibrotic scar following injury. The cryoinjury technique circumvents this problem: the injury promotes cell death and further formation of scar tissue, as observed in humans [20, 21, 23, 25, 26]. However, cryoinjury does not represent an ischemic damage followed by reperfusion injury, the common series of events occurring upon a myocardial infarction episode in humans [32, 70]. Moreover, unlike humans, the zebrafish is able to fully regenerate the heart following injury. Nevertheless, a deeper understanding of the early steps of the regeneration process may allow researchers to reconcile important differences. Despite these limitations, our analysis highlighted genes that are known to be involved in heart failure in other species, such as rodents, and even those known to play a crucial role in regulating cardiac function in humans, reinforcing thus the potential of our candidate genes as key regulators of heart regeneration.

## Conclusions

Because of its potential for translational research, we will continue investigating heart injury responses in the zebrafish using the cryoinjury model. We will expand the scope and depth of our investigations, including additional biological replicates at post-injury times as well as different control samples reflecting pre-injury or non-damaged states. As larger, high-quality datasets become available at our laboratory and elsewhere, we will be able to further assess the predictive potential of our network-based models.

In conclusion, we identified genes with potential critical roles in the response to cardiac damage in the zebrafish. Their transcriptional activities are reproducible in different *in vivo* models of cardiac injury. These findings motivate additional research to confirm and functionally characterize our set of candidate genes. These insights and the application of network-based computational models open new opportunities for cardiovascular translational research.

## Methods

### Animal procedure and cryoinjury model

All procedures were approved by the national authorities responsible for animal experiments in Luxembourg. Experiments were performed on wild-type adult zebrafish aged 10–11 months from the WIK strain (ZIRC, Eugene, OR, USA). Animals were maintained under standard laboratory conditions in the Zebtec Stand Alone system (Tecniplast) at a density of 3 fish/L, at 28°C with day/night light cycles of 14 h light/10 h dark.

Cryoinjury and hearts dissection were performed as previously described [24]. Briefly, fish were anesthetized in 0.04% tricaine (Sigma Aldrich, Bornem, BE) and placed ventral side up in a damp foam. A small incision was performed through the body wall and the pericardium, the pericardial sac was opened and the ventricle exposed by gently compressing the abdomen. The surface of the ventricle was frozen by applying a cryoprobe previously cooled in liquid nitrogen, until thawing was observed. Following surgery, fish were placed in a tank of fresh water, reanimated by gently pipetting water onto the gills and further housed under standard conditions. Sham operations consisted of opening the body wall and the pericardial sac, without touching the exposed ventricle. Animals were sacrificed 1 or 3 days after surgery by immersion in 0.16% tricaine (Sigma Aldrich, Bornem, BE). Hearts were dissected in PBS containing heparin and KCl and further used for histological staining or RNA extraction.

### Immunohistochemistry

Hearts were fixed overnight in PBS containing 4% paraformaldehyde, dehydrated and embedded in paraffin wax. Seven μm sections were cut on a Leica Microtome, mounted on superfrost slides and dried for 1 h at 45°C. Samples were deparaffinized in xylol, rehydrated in ethanol and washed in distilled water. Heat-induced epitope retrieval was performed in citrate buffer pH 6. Sections were blocked for 1 h in 5% BSA – 0.5% tween 20. Primary anti-tropomyosin antibody was CH1 (DSHB) used at a dilution of 1/20. Secondary antibody was Alexa Fluor®568 goat-anti mouse IgG (1/300; Molecular Probes). TUNEL staining was performed using the *in situ* Cell Death Detection Kit, Fluorescein (Roche), according to the manufacturer’s instructions. Nuclei were stained with DAPI. Pictures were acquired using a confocal fluorescence microscope (Zeiss Laser Scanning Microscope LSM 510).

### Independent validation: RNA extraction, integrity and reverse transcription

Three to four heart ventricles were pooled per sample in TRIzol (Invitrogen, Carlsbad, CA) and stored at -80°C until extraction. Homogenisation of samples was performed with a Polytron® (Bohemia,USA). Aqueous phase was isolated with Phase lock gel-Heavy (5 Prime, Gaithersburg, MD), total RNA was precipitated with 100% isopropanol and purified using RNeasy Micro kit combined with an on-column DNase treatment following the manufacturer’s instructions (Qiagen, Valencia, CA). RNA quantity was assessed with a Nanodrop (Thermo Scientific, Wilmington, USA) and quality was evaluated with the Agilent 2100 Bioanalyzer (Agilent Technologies, Palo Alto, CA). RNAs used in the present study were of good quality and un-degraded (Ratio A260/A280 ≈ 2 and RIN ≥ 8).

500 ng of RNA were reverse transcribed into cDNA using the SuperScript III (Invitrogen, Carlsbad, CA) reverse transcriptase with the following protocol: RNAs were mixed with random primers, oligo (dT)12-18 and dNTPs in a total volume of 13 μl. Samples were heated to 65°C for 5 min and incubated on ice for at least 1 min. Then the 5X RT buffer, DTT, RNaseOUT and SuperScript III were added to a total volume of 20 μl. RT was allowed at 50°C for 60 min and was followed by enzyme inactivation at 70°C for 15 min. Final concentrations were: 100 ng of oligo(dT)12-18, 50 ng of random primers, 0.5 mM dNTPs, 50 mM Tris–HCl, 75 mM KCl, 3 mM MgCl2, 5 mM DTT, 40U of RNaseOUT and 200U of SuperScript III. To remove RNA complementary to the cDNA, 2U of *E. coli* RNaseH were added and samples were incubated at 37°C for 20 min. In each RT-PCR a no template control (no RNA in RT) was performed.

### Independent validation: qPCR experiments

cDNAs obtained from RT of RNA were diluted 10-fold and 4 μL were mixed with SYBR®Green Master Mix (Biorad, Nazareth, Belgium) to a final volume of 20 μL containing 300nM of each primer. Amplification was carried out in the CFX96 thermal cycler (BioRad) under the following conditions: heating for 3 min at 95°C, 40 cycles of denaturation for 30 s at 95°C, followed by an annealing/extension for 1 min. After each run a melting curve analysis was performed, ramping from 55°C to 95°C in 20 min. A negative control without cDNA template was run in every assay and measures were performed in duplicates.

Primers were designed with the Beacon Designer Pro 8.10 software (Premier Biosoft, Palo Alto, USA), flanking an intron. Specificity was assessed using the NCBI BLAST tool [71], melting curves were performed in each assay and gene-specific amplification was confirmed by a single band in 4% E-Gel® (Life technologies). Data analysis normalization was carried out against three reference genes; *ef1a, rpl13a, tuba1* and expression levels were calculated using the CFX manager 3.0 software (Biorad) via the delta-delta Cq method, taking into account the calculated amplification efficiency for each primers pair. See Additional file 5 for MIQE checklist [72] and details of qPCR experiments.

### Data pre-processing and differential expression analysis

As we were intended to use already pre-processed microarray data, no normalization or probe summarization was performed in addition to those used in [29, 40]. In order to increase the power of statistical testing for detecting differential expressed genes, we performed pre-filtering of features removing those, which never show expression over 6 in log2 scale. The cleaned dataset was then analysed by limma package of R/Bioconductor [73] as it was described before [74, 75]. Finally, a list of features significantly regulated at each time point was obtained using the same “limma” model and a set of contrasts, comparing time point samples versus controls. To control for false discovery rates (FDR), we used the Benjamini–Hochberg’s adjust of P-values. To report changes in gene expression we used standard heatmaps of R/Bioconductor.

### Gene association network models

We generated and analysed gene co-expression networks with different computational methodologies: Clustering with Overlapping Neighborhood Expansion (ClusterONE) [37], inference of gene regression networks with model trees (RegNet) [38], and the Weighted Gene Co-Expression Network (WGCNA) algorithm [39]. The capacity of these techniques to produce biologically informative results have previously been reported in different biomedical research applications. These techniques allowed us to identify: networks clusters, highly connected genes, and genes that can be used as estimators of the expression values of other genes in the dataset.

The Clustering with Overlapping Neighborhood Expansion (ClusterONE) algorithm identifies highly cohesive network clusters (modules) through a 3-step process [37]. First, starting from a seed node, ClusterONE implements a greedy search of candidate groups showing high cohesiveness. This procedure is started from different seed nodes and leads to the formation of multiple, and possibly overlapping, clusters. Next, ClusterONE merges clusters with significant overlaps according to a pre-defined threshold. In the final step, candidate modules that do not meet user-defined scores (minimum number of nodes and module density) are removed from the list of candidate modules. Here we used ClusterONE’s plugin (v1.0) for Cytoscape (v2.8.3) [76] with a minimum number of nodes of 5, “Auto” minimum density, unweighted clustering, node penalty = 2, haircut threshold = 0; overlap threshold = 0.8.

RegNet [38] identifies transcriptional association networks in three steps. In the first step, an unsupervised learning algorithm analyses each gene as a target by taking into account the remaining genes as inputs to a mathematical model that estimates the expression value of that gene. The mathematical model consists of several linear models spread over separate areas of the search space, i.e. optimal partitions of gene expression samples. Each linear model represents localized similarities between specific groups of genes. Moreover, the algorithm builds linear models under all samples (global similarity) if the optimal subspace is defined by the complete set of gene expression samples. We have used M5’ model tree algorithm [77] to build the mathematical model for each target gene. In the second step, RegNet extracts, as hypothetical evidences of gene-gene association, the linear dependency that exists between the target gene and every gene involved in the linear models of the target gene. This step only includes those mathematical models that have a relative error less than a threshold value. The third step involves building a graph model of gene co-expression network by assessing the significance of the set of hypothetical evidences using the Benjamin-Yekutieli procedure for the control of false discovery rate. In our analysis RegNet was applied with a threshold value theta = 30%, i.e. the model trees with relative error greater than theta were removed, and statistical significance at 0.05.

Module identification was also performed using Weighted Correlation Network Analysis (WGCNA) [39]. With WGCNA, weighted correlations are calculated between genes based on the gene’s expression in the different samples; a weighted correlation is a correlation raised to a certain power (called the soft thresholding power). Weighted correlations are used as they are more stringent for detecting true correlations, as they will favor higher correlations over lower correlations (which can often occur due to chance). In WGCNA, the weighted correlations are used to create a specific type of network (a topological overlap network). This network is based on network topology rather than direct correlations between genes: genes are strongly linked together in this network if they share many correlated neighbours with each other. This topological overlap network is then analysed to identify network modules of genes that are strongly linked together. Identifying network modules from the gene expression in this way focuses on groups of genes that are predicted to be functionally related based only on the gene expression data. WGCNA was performed on genes that were significantly differentially expressed between at least two conditions across all samples (FDR < 0.05). The network was constructed by calculating an adjacency matrix using a soft-thresholding power of 10 and Spearman correlation using pairwise complete observations. A topological overlap matrix (TOM) was then calculated from the adjacency matrix, converted to distances, and clustered by hierarchical clustering using average linkage clustering. Modules were identified with dynamic tree cut with minimum module size = 20, using the hybrid method. Module eigengenes were calculated and similar modules were merged together using a module eigengene distance of 0.15 as the threshold.

## Electronic supplementary material

Additional file 1: **Dynamic visualization of co-expression network at different times (PDF) and underlying data (Excel file).** In the network visualizations: nodes are color-coded to reflect Log2FC values (in relation to control), with scales indicated for each time-specific state. (ZIP 2 MB)

Additional file 2: **Detailed, higher resolution view of Figure** 2 **C.** (PDF 186 KB)

Additional file 3: **Immunohistochemistry on sagittal sections of sham-operated and cryoinjured hearts recovered one day post-surgery.** A – D: Staining is performed with an antibody against tropomyosin (red). Note the reduced staining of tropomyosin in the injured area of the ventricle compared to sham. E –H: TUNEL assay on hearts recovered one day post-surgery detects a high amount of apoptotic cells (green) in the cryoinjured area while no staining is visible in uninjured ventricles. Nuclei are stained with DAPI (blue). A/C: costaining DAPI/tropomyosin; B/D: staining of tropomyosin alone. E/G: costaining DAPI/TUNEL; F/H; TUNEL staining alone. Scale bar is 100 μm. A: atrium; B: bulbus arteriosus; V: ventricle; IA: injured area. Sham: sham-operated heart recovered one day post-surgery; 1dpi: cryoinjured heart recovered at day-1 post-injury. (PNG 9 MB)

Additional file 4: **Changes in (log2) expression of candidate genes in independent qPCR data validation with corresponding standard errors (calculated based on expression variability of 3 biological replicates for experimental and control conditions).** (A) 1 day after injury vs sham-1d and (B) 3 days after injury vs sham-3d. Central node in Figures 6 and 7, *ptgis*, is marked yellow. (TIFF 74 KB)

Additional file 5: **MIQE checklist, including details of qPCR experiments.** Sheet 1: MIQE, Sheet 2: A-Primers B- Normalization data analysis information. (XLS 76 KB)
