# Supplementary figures and images for: Transcriptional response to cardiac injury in the zebrafish: systematic identification of genes with highly concordant activity across in vivo models
Source: BMC Genomics. 2014 Oct 3;15(1):852. doi: 10.1186/1471-2164-15-852 (PMC4197235; doi:10.1186/1471-2164-15-852)

Day 1

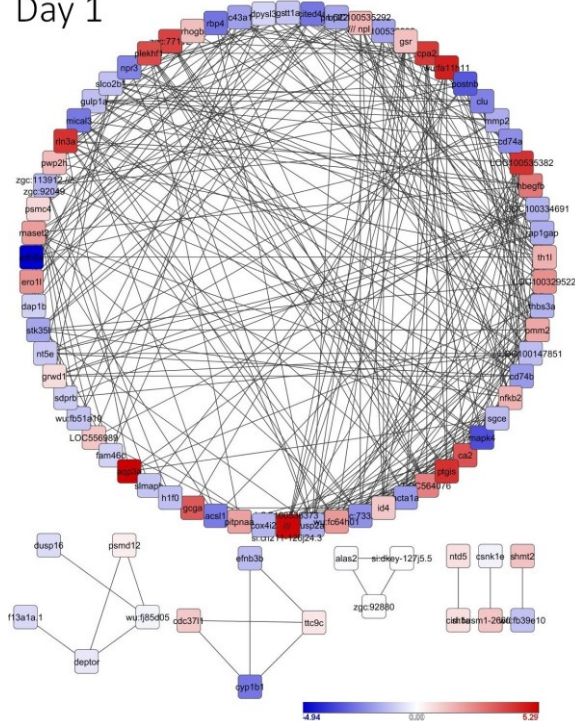

Day 3

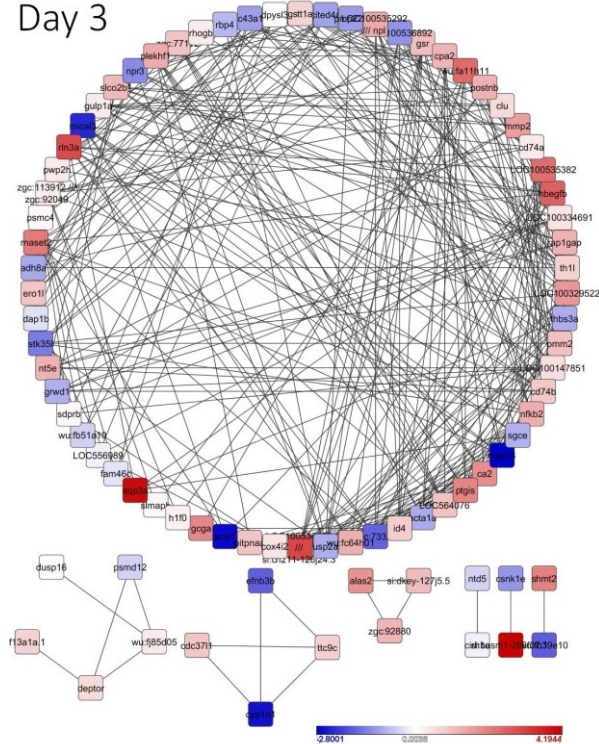

Day 5

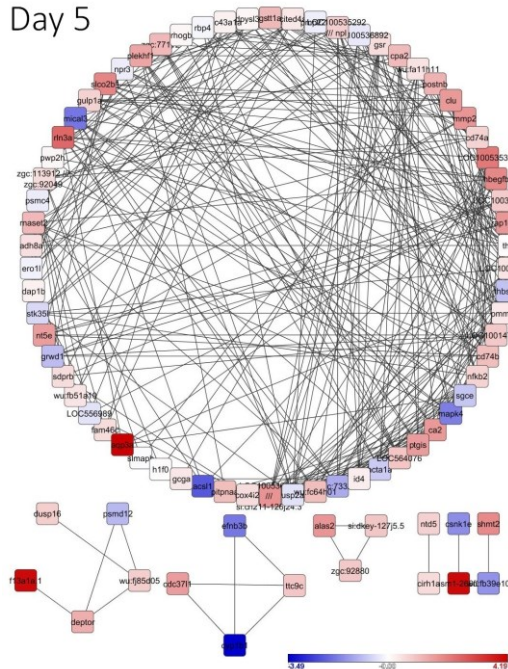

Day 7

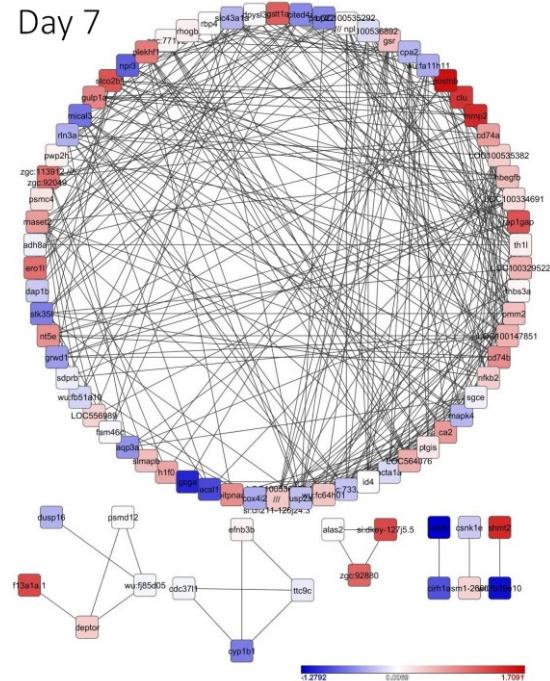

Supplement: Supplementary file 1 — Additional file 1: Dynamic visualization of co-expression network at different times (PDF) and underlying data (Excel file). In the network visualizations: nodes are color-coded to reflect Log2FC values (in relation to control), with scales indicated for each time-specific state. (ZIP 2 MB) [file 12864_2014_6534_MOESM1_ESM.zip › time-specificVisuals.pdf]

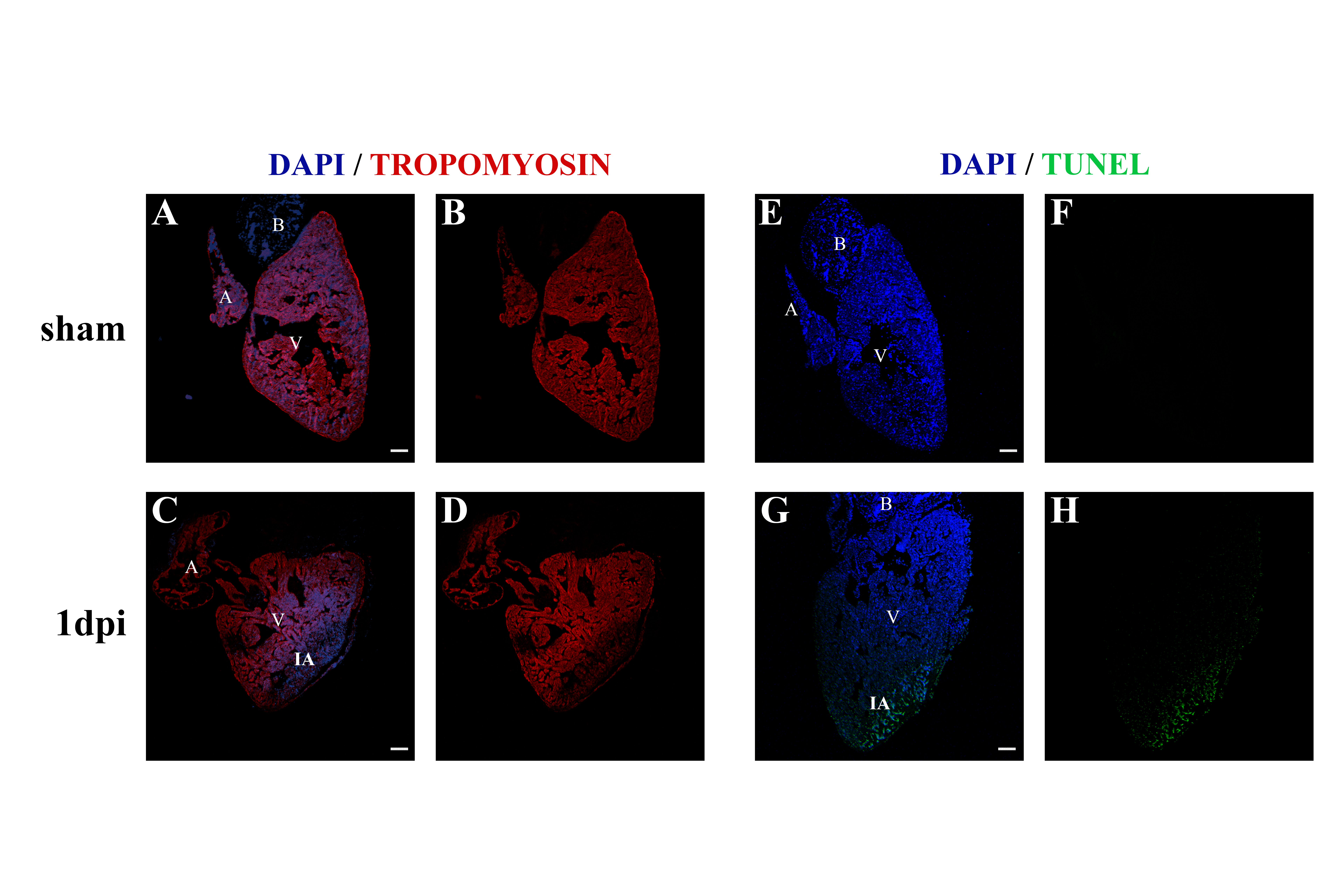

Supplement: Supplementary file 3 — Additional file 3: Immunohistochemistry on sagittal sections of sham-operated and cryoinjured hearts recovered one day post-surgery. A – D: Staining is performed with an antibody against tropomyosin (red). Note the reduced staining of tropomyosin in the injured area of the ventricle compared to sham. E –H: TUNEL assay on hearts recovered one day post-surgery detects a high amount of apoptotic cells (green) in the cryoinjured area while no staining is visible in uninjured ventricles. Nuclei are stained with DAPI (blue). A/C: costaining DAPI/tropomyosin; B/D: staining of tropomyosin alone. E/G: costaining DAPI/TUNEL; F/H; TUNEL staining alone. Scale bar is 100 μm. A: atrium; B: bulbus arteriosus; V: ventricle; IA: injured area. Sham: sham-operated heart recovered one day post-surgery; 1dpi: cryoinjured heart recovered at day-1 post-injury. (PNG 9 MB) [file 12864_2014_6534_MOESM3_ESM.png]

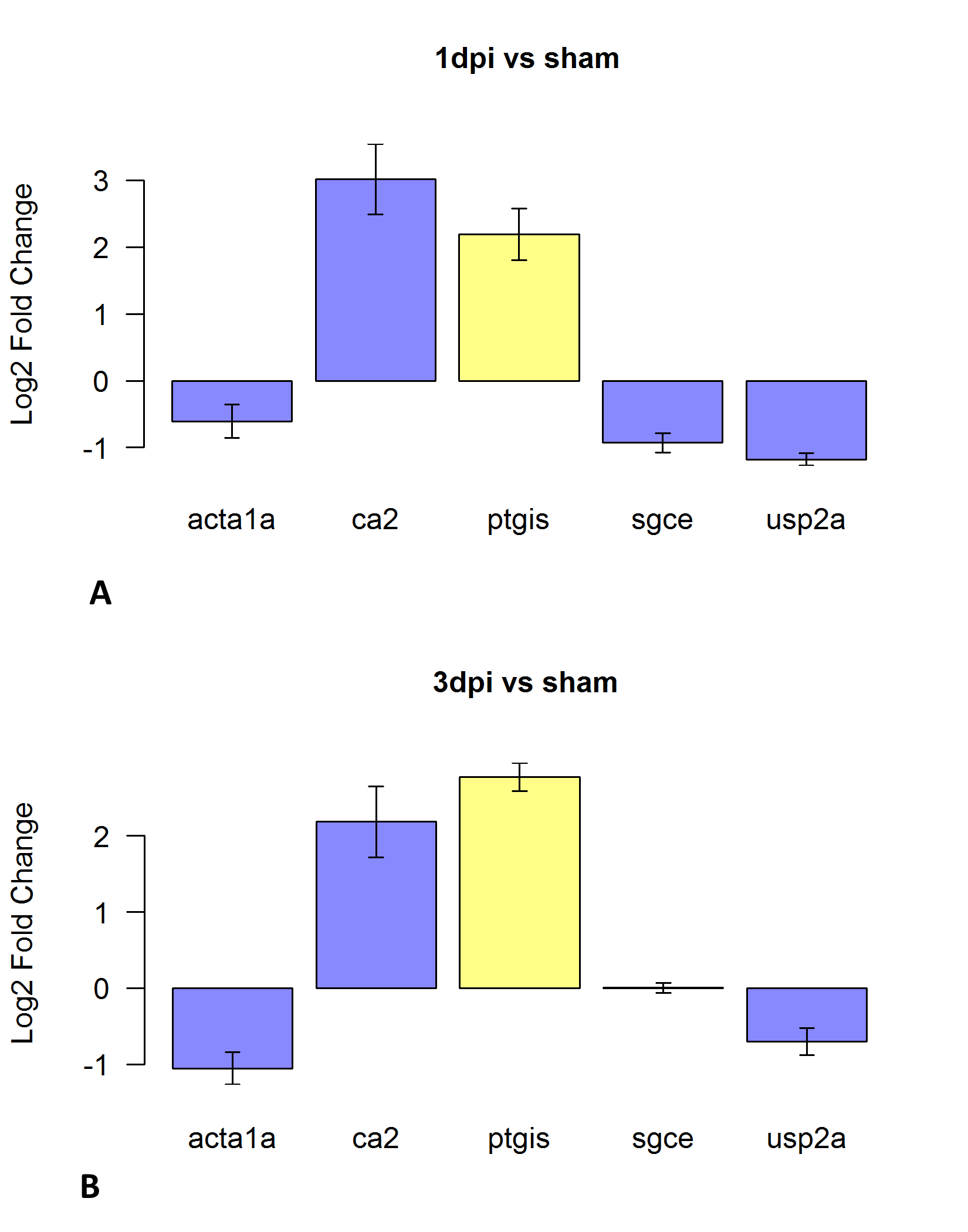

Supplement: Supplementary file 4 — Additional file 4: Changes in (log2) expression of candidate genes in independent qPCR data validation with corresponding standard errors (calculated based on expression variability of 3 biological replicates for experimental and control conditions). (A) 1 day after injury vs sham-1d and (B) 3 days after injury vs sham-3d. Central node in Figures 6 and 7, ptgis, is marked yellow. (TIFF 74 KB) [file 12864_2014_6534_MOESM4_ESM.tiff]
