# Supplementary material for: Transcriptional response to cardiac injury in the zebrafish: systematic identification of genes with highly concordant activity across in vivo models
Source: BMC Genomics. 2014 Oct 3;15(1):852. doi: 10.1186/1471-2164-15-852 (PMC4197235; doi:10.1186/1471-2164-15-852)
Supplement: Supplementary file 2 — Additional file 2: Detailed, higher resolution view of Figure 2 C. (PDF 186 KB) [file 12864_2014_6534_MOESM2_ESM.pdf]

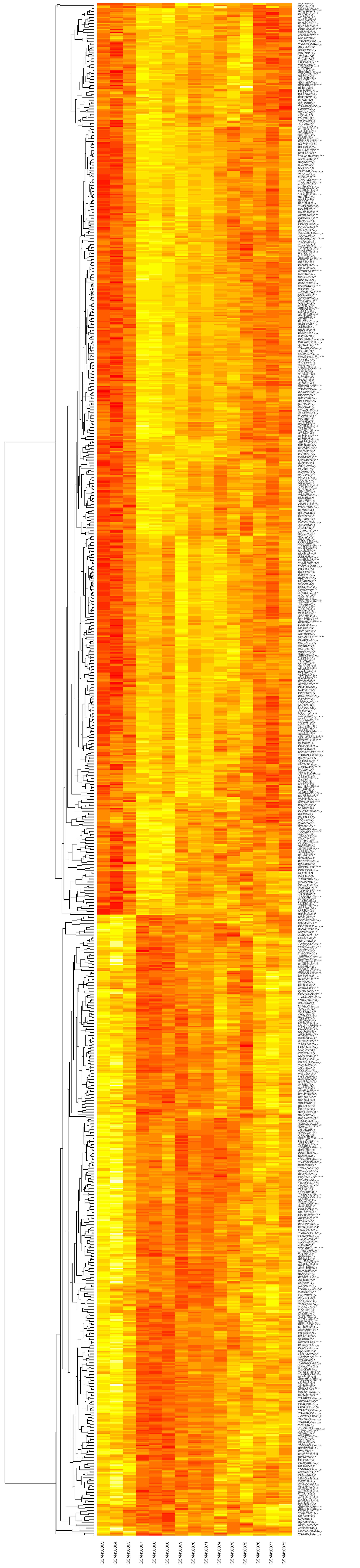
GSM450363
GSM450364
GSM450365
GSM450367
GSM450368
GSM450366
GSM450369
GSM450370
GSM450371
GSM450374
GSM450373
GSM450372
GSM450376
GSM450377
GSM450375
